# Supplementary figures and images for: A shear stress micromodel of urinary tract infection by the Escherichia coli producing Dr adhesin
Source: PLoS Pathog. 2020 Jan 9;16(1):e1008247. doi: 10.1371/journal.ppat.1008247 (PMC7004390; doi:10.1371/journal.ppat.1008247)

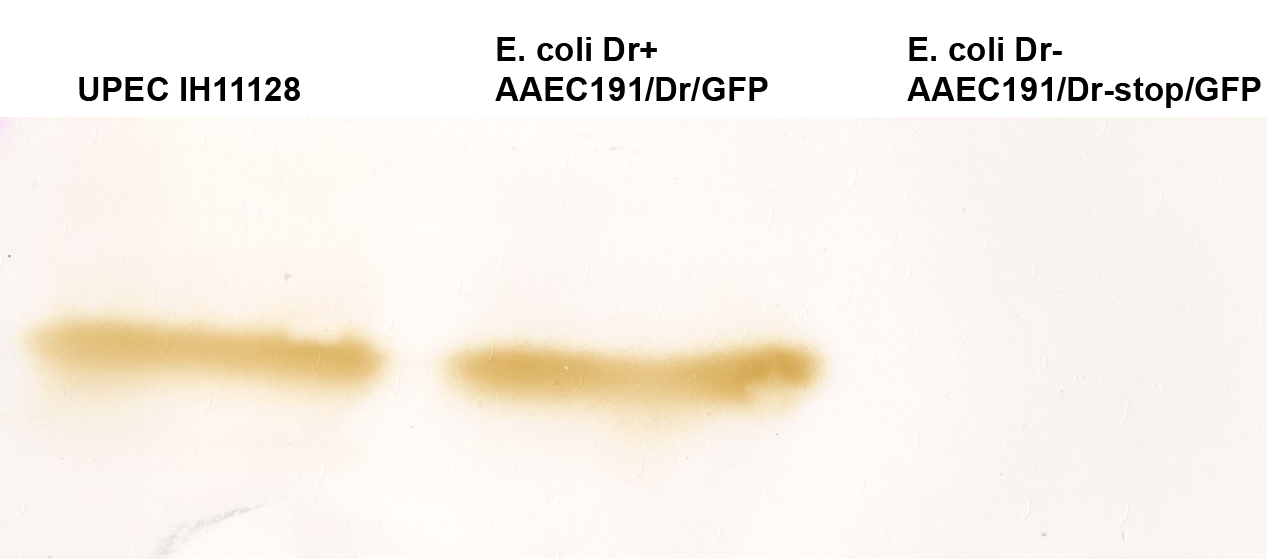

Supplement: S1 Fig — Representative Western blotting analysis of fimbrial fractions isolated from the: IH11128 UPEC clinical strain, laboratory Dr+ AAEC191/Dr/GFP strain and laboratory Dr- AAEC191/Dr-stop/GFP strain. Prior to isolation of fimbrial fractions, the overnight bacteria cultures were centrifuged and resuspended in a PBS to OD600 of 1.0. Western blotting was performed using primary rabbit anti-Dr and secondary goat anti-rabbit antibodies labeled with horseradish peroxidase. The relative concentration of DraE in the fimbrial fraction isolated from Dr+ and Dr- bacteria was determined by densitometry analysis, with the IH11128 UPEC strain used as a reference (100%). The experiment was repeated three times. The laboratory Dr+ strain produces Dr fimbriae at a level of 105 ± 12% relative to IH11128. For the Dr- strain no signal corresponding to Dr fimbriae was recorded. (TIF) [file ppat.1008247.s001.tif]

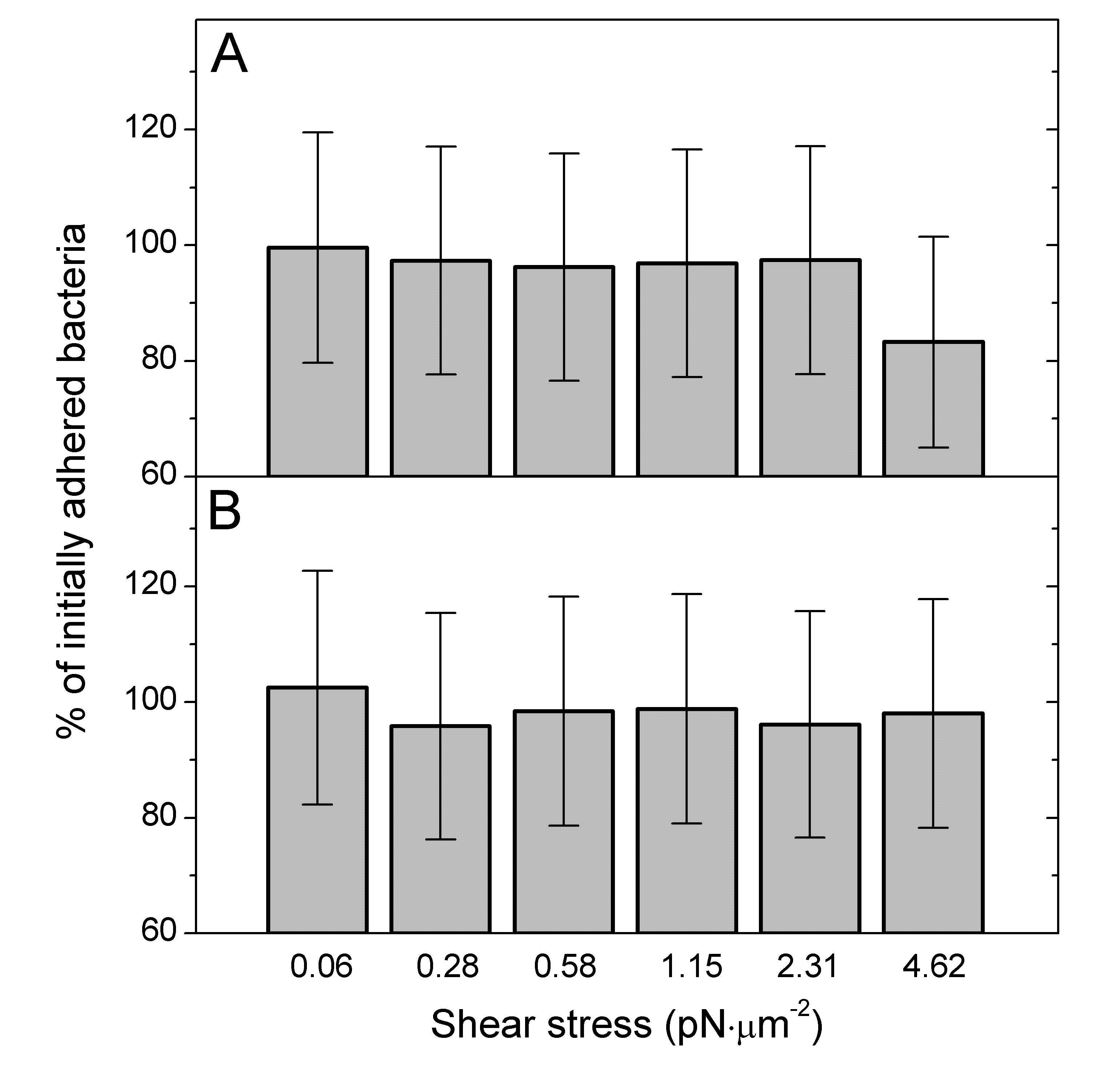

Supplement: S2 Fig — (A) and (B): the flow detachment experiment without and with 2-minute pauses between subsequent flow velocity steps, respectively; see Fig 2D caption for experimental details. The figure shows the fraction of bacteria that remain bound at the end of each 264-second flow step, determined as the difference between bacteria count at the start and end of each flow step. The error bars give 95% confidence intervals of 12 replicates. (TIF) [file ppat.1008247.s002.tif]

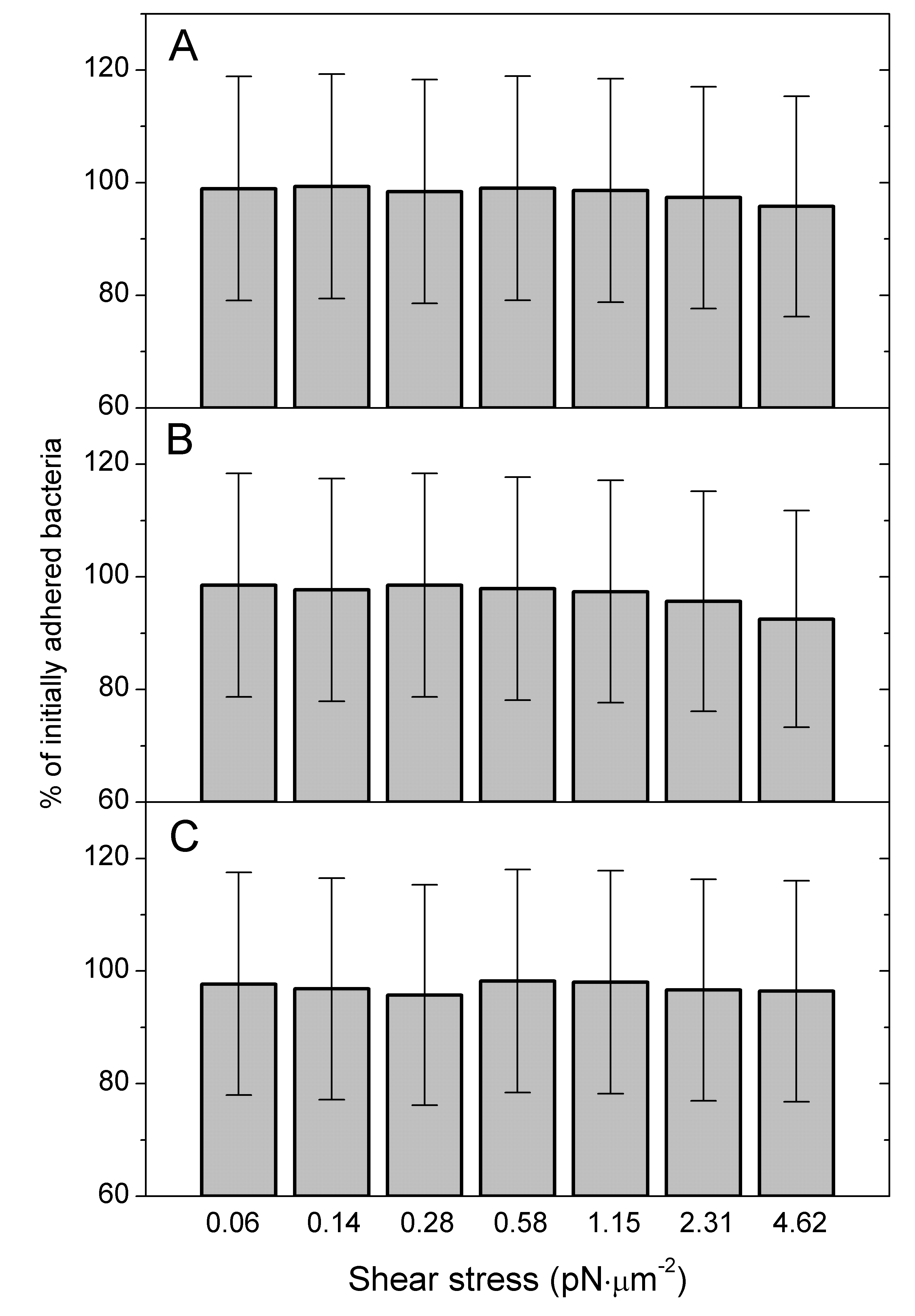

Supplement: S3 Fig — (A), (B) and (C): the flow detachment experiment using flow medium supplemented with 300, 600 and 2000 μM Cm, respectively; see Fig 3D for experimental details. The figure shows the fraction of bacteria that remain bound at the end of each 180-second flow step, determined as the difference between bacteria count at the start and end of each flow step. The error bars give 95% confidence intervals of 12 replicates. (TIF) [file ppat.1008247.s003.tif]

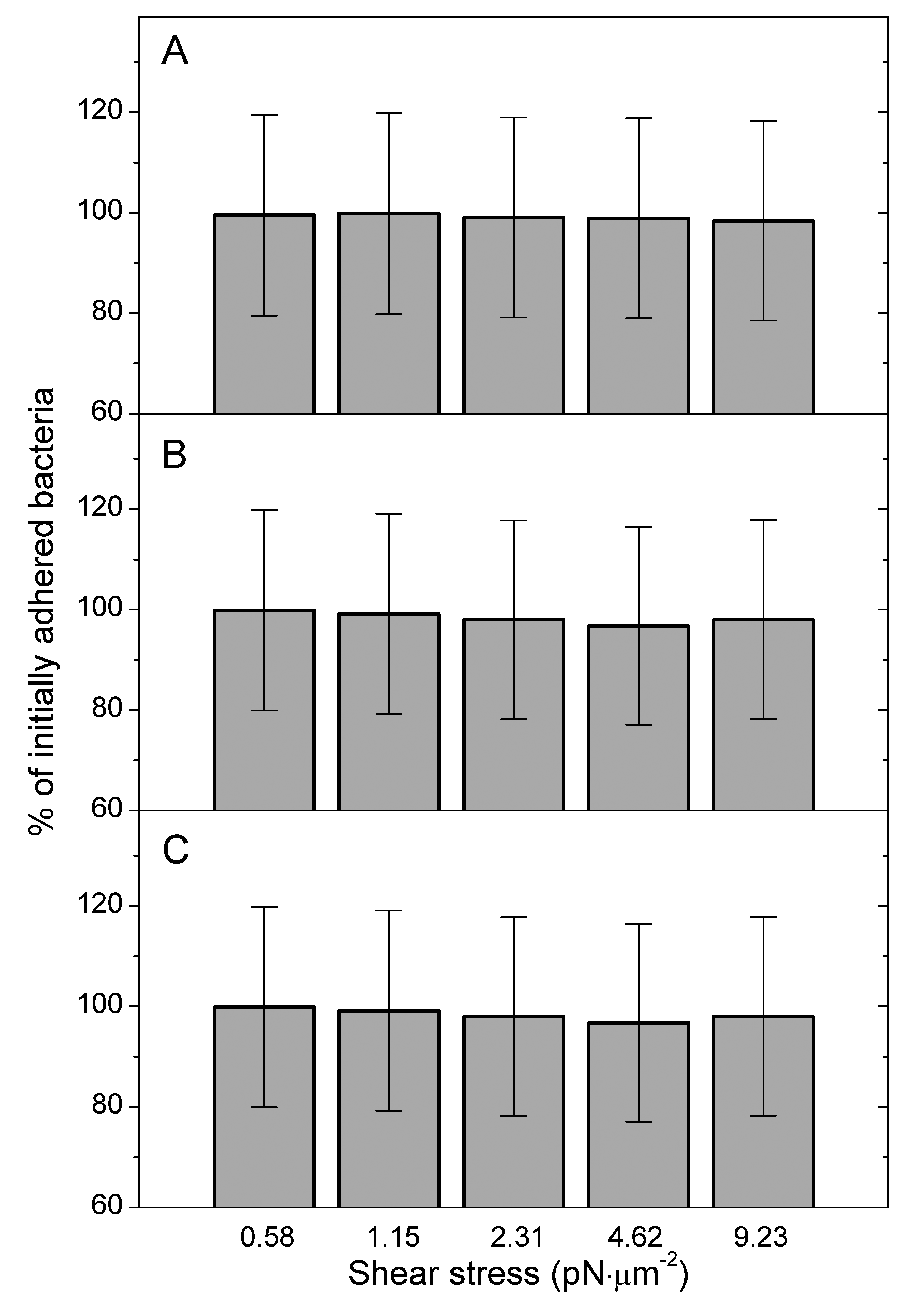

Supplement: S4 Fig — (A), (B) and (C): the flow detachment experiment using dishes coated with 20, 2 or 0.2 μg ml-1 human type IV collagen; see Fig 4C for experimental details. The figure shows the fraction of bacteria that remain bound at the end of each 192-second flow step, determined as the difference between bacteria count at the start and end of each flow step. The error bars give 95% confidence intervals of 12 replicates. (TIF) [file ppat.1008247.s004.tif]

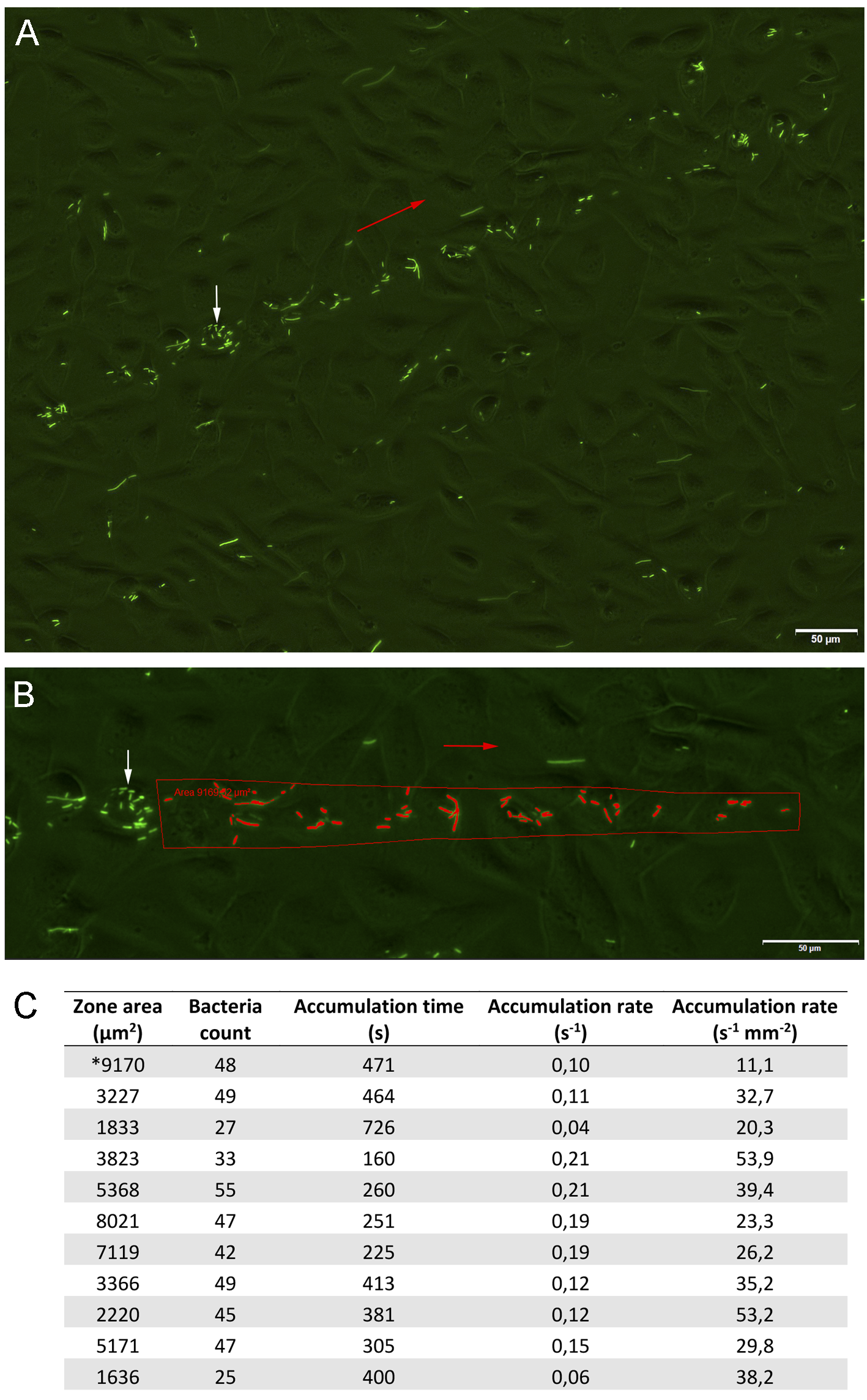

Supplement: S5 Fig — Dr+ E. coli were washed through a flow chamber with an overgrown layer of T24 cells with 24 ± 8 budding cells in the field of view, at a shear stress of 0.42 pN μm-2 for 20 minutes. (A): increased adherence zones of Dr+ bacteria, characterized by well-defined boundaries. (B): zone area was determined using ‘Measure’ command, and the final number of bound bacteria in a given zone was determined using the ‘Count and Measure’ package of Olympus cellSens software. Time of zone formation was counted from the moment of attachment of the first until the attachment of the last bacteria. In panels (A) and (B) white and red arrows mark the positions of the budding cell and direction of flow, respectively. Bars correspond to 50 μm. Table (C) represents zone areas (5% accuracy), the number of accumulated bacteria (3% accuracy) and time of zone formation for 11 well-defined adherence zones. From these data, the rate of bacteria accumulation in zone per second and per second per mm2 was calculated with an error of 3% and 6%, respectively. *—denotes data for adherence zone presented in panels (A) and (B). (TIF) [file ppat.1008247.s005.tif]
